# Middle Cerebral Artery Dissection: A Report of Two Cases With Treatments and Outcomes

**DOI:** 10.7759/cureus.39632

**Published:** 2023-05-29

**Authors:** Giulio Papiri, Stefano Bruni, Emanuele Puca, Sandro Sanguigni, Matteo Marcucci

**Affiliations:** 1 Neurology Unit, Ospedale Provinciale Madonna del Soccorso, San Benedetto del Tronto, ITA; 2 Interventional Neuroradiology, Ospedale Regionale Umberto I, Ancona, ITA; 3 Neurology Unit, Ospedale Provinciale Madonna del Soccorso, San Benedetto Del Tronto, ITA; 4 Radiology Unit, Ospedale Provinciale Macerata, Macerata, ITA

**Keywords:** mca stroke, diffusion weighted imaging (dwi), cerebral digital substraction angiography, middle cerebral artery dissection, young onset stroke

## Abstract

In the present report, we describe two cases of right-sided M1 segment middle cerebral artery dissection in a 51-year-old Asian female and in a 28-year-old Caucasian male patient with no previous history of ischemic stroke or known intracranial atherosclerosis presenting with acute unilateral headache progressing to severe multifocal hemispheric infarction with nearly complete one-sided motor paralysis. In both patients, a middle cerebral artery dissection was detected on angiography; they were given exclusively medical therapy: patient 1 was not eligible to reperfusive therapies and was treated with a three-month course of acetylsalicylic acid and clopidogrel combined with low-dose enoxaparin, while patient 2 was initially treated with intravenous alteplase with no hemorrhagic complications and was later shifted to single antiplatelet therapy. Despite an initial worsening of clinical severity and an extensive ischemic lesion in both patients, neurologic function improved over time, eventually allowing recovery of unaided gait. Therefore, in the absence of signs of hemorrhage, intravenous thrombolysis or dual antiplatelet regimens could be considered in strokes related to middle cerebral artery dissection.

## Introduction

Intracranial artery dissection is a known cause of juvenile ischemic stroke but is a rare etiology of stroke in the adult population. It has been associated with 20% of strokes in young adults, is more frequent in Asian ethnicities, and may occur in the presence, or most frequently in the absence, of intracranial atherosclerosis [1]. A few cases have been described in temporal association with head trauma. Given the rarity of the condition, current treatment recommendations rely mostly on observational data [2,3].

In intracranial artery dissections, a primary vessel wall rupture is thought to lead to the formation of an intimal flap followed by a hematoma, which might extend over time in a longitudinal or radial trajectory, reducing downstream blood flow, while also causing a local thrombus formation, with potential embolic consequences [1,2].

In concordance with the degree of blood flow impairment, clinical severity may range from a mild headache to massive, life-threatening ischemic infarction, as in large vessel occlusion syndromes [4]. A worse prognosis has been described in more extensive dissections, whereas wall rupture is thought to progress to aneurysm formation, which is a lesion more prone to subarachnoid hemorrhage (SAH) [1,5].

Diagnosis mainly relies on clinical suspicion and often requires an angiographic confirmation [2]. As the pathogenesis of intracranial vessel dissections involves heterogeneous mechanisms, it has been suggested that Transcranial Color Doppler sonography (TCCD) might be a valuable asset for diagnostics as well for prognostic evaluation, being not only able to identify vessel steno-occlusion but also to characterize dynamic aspects such as microemboli signals, collateral circulation, and vasospasm [6,7].

In the following report, we present two cases of M1 middle cerebral artery dissection with extensive infarction and disabling neurological symptoms in which neither patient developed hemorrhagic complications. Patient 1 was treated, despite the presence of a large infarction, with a dual antiplatelet regimen, while patient 2 was treated with alteplase. In both patients, medical therapy was sufficient to achieve clinical stabilization.

## Case presentation

Case 1

A 51-year-old female patient of Indian ancestry was referred to the Emergency Department (ED) 24 hours after the onset of headache and left-sided hemiparesis which was initially intermittent, but later progressed and was continuously present. The patient described an accidental fall three days before symptom onset. Her past medical history was remarkable for arterial hypertension and nontoxic multinodular goiter.

On neurologic evaluation, she was found to be alert, with partial horizontal gaze paralysis, hemi-inattention, neglect, and left-sided weakness (complete upper limb paralysis, lower limb strength 3/5). Her NIHSS score was 11. At the ED, a head computed tomography (CT) scan showed an initial hypodensity in the territory of the right middle cerebral artery. A subsequent CT angiography scan showed images compatible with a right intracranial carotid artery stenosis and a left-sided 3 mm middle cerebral artery aneurism.

**Figure 1:**
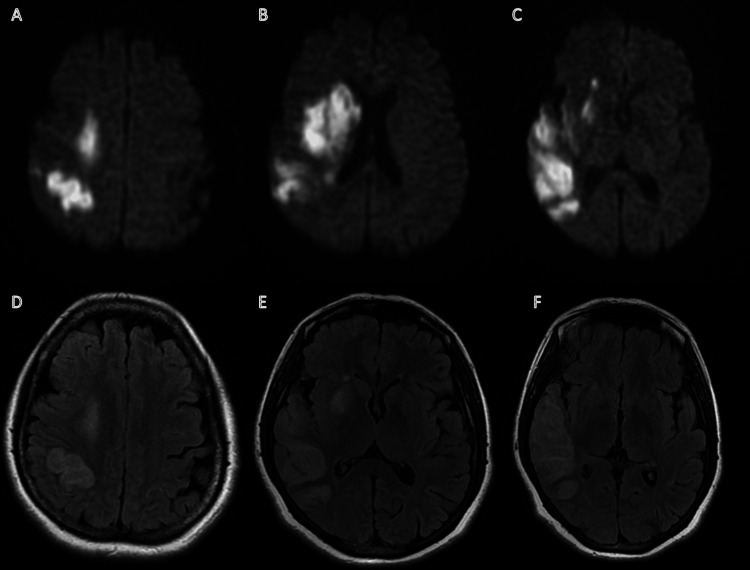
MRI study from patient 1 Panels A-B-C: DWI sequences showing multiple hyperintense foci in the right primary motor cortex (A), in the deep gray and white matter of the frontal lobe (B) and in the temporal lobe (C). Panels D-E-F: FLAIR sequences showing the corresponding ischemic areas.

Thrombolytic therapy was not performed given the time since symptom onset and the presence of an ischemic lesion. The patient was subsequently treated with i.v. Acetylsalicylic acid (ASA) 300 mg and was admitted to the local stroke unit. Over the following 48 hours, left-sided weakness progressed to hemiplegia, and on a subsequent MRI exam, a large acute, multifocal infarction in the right MCA territory was detected (Figure 1). Enoxaparin 40 mg OD was prescribed. A vasculitis panel (ANA, anti-ENA, lupus anticoagulant, c-ANCA, p-ANCA, anti-CCP antibodies) was negative. Alpha-galactosidase enzyme activity was within the normal range. No further diagnostic workup was performed.

**Figure 2:**
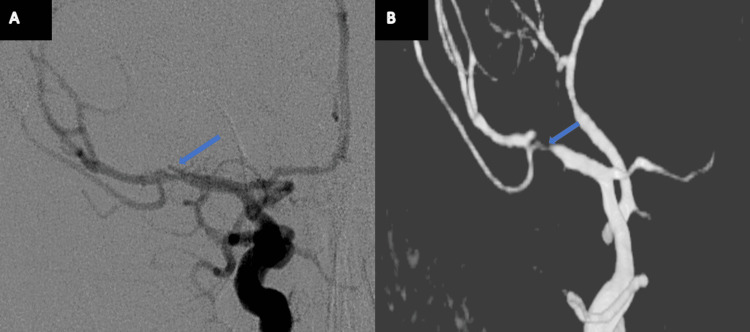
DSA study from Patient 1 Panels A-B: DSA scan (A) and reconstruction (B) showing a right-sided MCA M1 segment dissection (arrow) immediately followed by a downstream aneurysm, after vessel branching.

Transcranial Color Doppler (TCCD) evaluation revealed turbulent flow with increased velocity in the right MCA M1 segments, suggesting intracranial vascular abnormalities. Digital subtraction angiography (DSA) revealed the presence of right-sided middle cerebral artery M1 dissection coexisting with a 3.5 mm long downstream M3 segment aneurism (Figure 2A, 2B).

After a collegial discussion, given the partial recanalization of the vessel, no indication for endovascular or surgical therapy was given. Dual antiplatelet therapy (acetylsalicylic acid 100 mg, clopidogrel 75 mg) was started without a clopidogrel loading dose, in addition to low-dose enoxaparin (40 mg).

The patient subsequently experienced a good recovery of lower limb strength and was transferred to a rehabilitation facility. Over the next two months, she recovered her walking capabilities and some upper limb movements. Enoxaparin was continued for one month, while clopidogrel and acetylsalicylic acid were continued in association for three months. At the end of the three months, only clopidogrel was left.

At hospital discharge, lower limb strength was 4/5, and upper limb strength was 3/5, with moderate spastic hypertonia. The Modified Rankin Score on discharge was 3.

Case 2

A 28-year-old male patient of Caucasian ethnicity was referred to the ED after the acute onset of headache and left-sided hemiparesis. He had no known cardiovascular risk factors or family history suggestive of juvenile cerebrovascular accidents. His only remarkable comorbidity was episodic migraine without aura, and not on prophylaxis therapy.

On neurologic examination, he showed a 3/5 left-sided motor weakness, and was alert and anosognosic with no overt gaze or sensory deficit. He was given a head CT scan completed with epiaortic and intracranial CT angiograms, which showed no acute hypodensities or large vessel occlusion. His NIHSS score was 5. He was treated with 0.9 mg/kg intravenous alteplase, without significant variations in his neurologic status.

**Figure 3:**
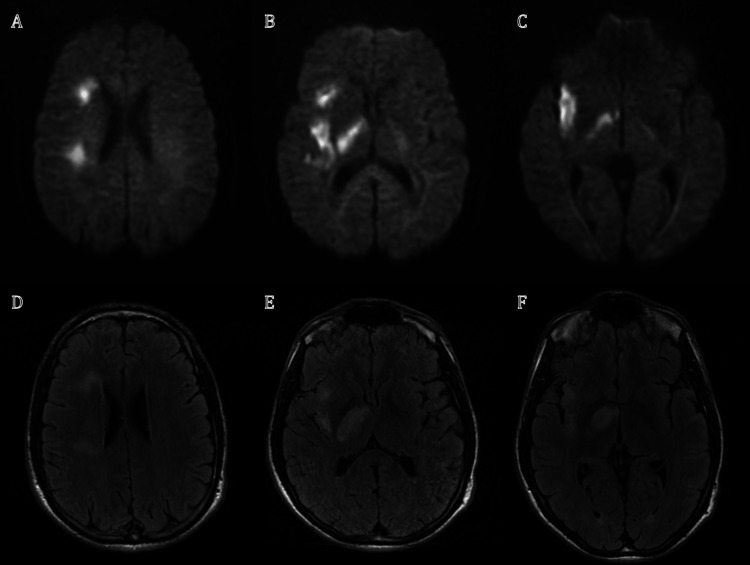
MRI study from patient 2 Panels A-B-C: DWI sequence showing hyperintense signal in the right internal, external capsules (A-B) and in the deep white matter of the frontal lobe (C). Panels D-E-F: Corresponding FLAIR sequences showing the extension of the ischemic lesion.

Over the subsequent 24 hours, his NIHSS score remained stable. Brain MRI detected a large right-sided infarction involving the internal capsule and the deep frontal lobe white matter, suggestive of proximal middle cerebral artery occlusion (Figure 3). He was treated with i.v. ASA (250 mg) due to the absence of hemorrhagic lesions.

Two days after symptom onset, his motor deficit worsened to 1/5 in both limbs; dysarthria and hemi-inattention appeared. His NIHSS score increased to 12. TCCD revealed flow abnormalities in the right intracranial carotid and middle cerebral artery while excluding a patent foramen ovale.

Low-dose enoxaparin (40 mg) was added to ASA. A subsequent epidural and intracranial CTA showed no significant variations in the ischemic volume but detected right-sided intracranial carotid stenosis followed by an increased ipsilateral M1 segment vascularization signal, suggesting intracranial vessel pathology.

**Figure 4:**
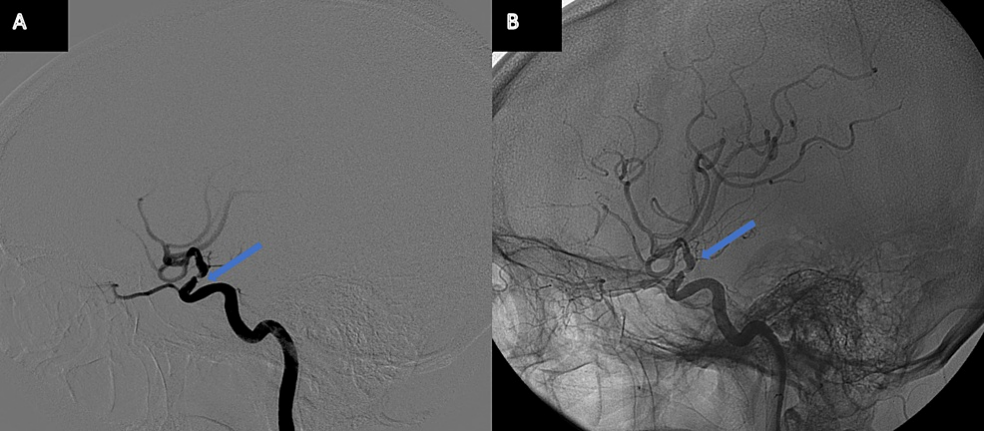
DSA study from Patient 2 Panel A: Angiogram showing a right-sided intracranial carotid artery stenosis. Panel B: Angiogram showing an intracranial carotid artery stenotic trait followed by a downstream M1 segment dissection (arrow).

A subsequent cerebral DSA confirmed the presence of a right-sided clinoid carotid segment stenosis associated with M1 middle cerebral artery dissection (Figure 4A, 4B). After a multidisciplinary consultation, given the partial vessel patency, no indication for endovascular treatment was given.

An immunologic panel (ANA, anti-ENA, lupus anticoagulant, c-ANCA, p-ANCA, anti-CCP antibodies) was negative. Alpha-galactosidase enzyme activity was within normal ranges as well. No further diagnostic work-up was carried out.

Over the following week, disability partially improved; hemi-inattention and dysarthria resolved, while motor deficits remained stable. Brain MRI revealed no significant variation in the ischemic lesion volume. Enoxaparin was suspended after one month when satisfactory trunk control was obtained.

The patient was transferred to a rehabilitation facility, and over two months, he completely recovered his motor deficit. On discharge, left-sided upper limb strength was 5/5, and lower limb strength was 5/5 with no residual hypertonia. The Modified Rankin Score on discharge was 1.

## Discussion

The presented cases show how the diagnosis and management of middle cerebral artery dissections might be challenging. At the onset, symptoms might be heterogeneous in their severity and develop in an acute, subacute, or fluctuating fashion [8]. Medical treatment should be evaluated on an individual basis since the pathophysiology in this setting is complex and might encompass multiple mechanisms at once, while the risk of hemorrhagic complications might pivot on several factors, although a longitudinal and transmural extension of the vessel wall rupture is considered the most relevant [1,9]. Pathogenesis of strokes in intracranial dissections is thought to involve either thromboembolic (artery-to-artery embolism, intramural hematoma expansion with vasa vasorum and perforator vessel occlusion) or hemodynamic mechanisms (hypoperfusion in “watershed” territories); furthermore, intramural hematoma expansion may damage an intracranial artery sufficient enough to produce a subarachnoid hemorrhage (SAH) [9]. It is also not completely understood which factors influence the vessel’s healing process, although the absence of an elastic lamina is thought to concur with wall vulnerability, hematoma formation, expansion, and development into intracranial fusiform aneurysms [9].

In consideration of its multifactorial pathogenesis, it is not well-known if, and at what time, medical treatment, i.e., antiplatelet or anticoagulant therapy, yields better results in terms of thromboembolic events or hemorrhagic risk with respect to surgical or endovascular treatment [10].

SAH has been linked to a worse prognosis, in terms of mortality and functional recovery. Endovascular (stenting) and surgical [11,3] (craniotomy with trapping, wrapping, clipping, extracranial-intracranial bypass, or resection) approaches have been preferred in scenarios where symptoms progress or recur despite medical therapy, or when there is evidence of rapid aneurysm growth, vessel closure, or subarachnoid bleeding [9]. Despite limited data, good functional outcomes (Modified Rankin Scale 0-2) have been observed in approximately 63-70% of Intracranial arterial dissections with SAH treated with endovascular or surgical procedures, compared to 35% in patients given only medical treatment [2].

In milder cases, with transient or stable neurologic deficits, anticoagulation or antiplatelet regimens have usually been preferred. At the moment, according to expert consensus, at variance from extracranial artery dissection, antiplatelet therapy is considered safer than anticoagulant therapy due to the concern for potential hematoma enlargement [2]. As for the hyperacute phase, it is not known whether thrombolysis might be beneficial when intracranial artery dissection is suspected, although some cases with good outcomes have been described in the literature [11]. On the other hand, as for Mechanical Thrombectomy (MT), in a small series of patients with ischemic stroke related to middle cerebral artery dissection, successful reperfusion has been achieved, although the procedure might carry a significant risk of fatal wall rupture [12]. The potential utility of endovascular treatment combined with intracranial stenting has also been suggested, although it should be decided on an individual basis due to the potential risk of perforator vessel occlusion [2]. 

In our small series, both patients were treated with combined antiplatelet and low-dose thromboprophylaxis, with clinical stabilization. In particular, patient 1 was started on dual antiplatelet therapy, despite the presence of an aneurysm, with no hemorrhagic complications, while patient 2 was treated with alteplase in the acute phase. We, therefore, suggest that even large infarctions related to middle cerebral artery dissection may benefit from aggressive medical therapy where baseline hemorrhagic risk is low and no overt signs of intracranial bleeding are detected. Furthermore, given the presence of vessel patency on TCCD, it could be speculated that the stabilization of MCA flow might have concurred with clinical stability. 

## Conclusions

In this study, we present two cases of juvenile acute ischemic stroke related to middle cerebral artery dissection. Clinical suspicion arose after both patients deteriorated after initial clinical stabilization, although in both cases, CT angiograms had not disclosed any large vessel occlusion. We, therefore, suggest that digital subtraction angiograms should be obtained as soon as possible in stroke in young adults as part of their routine diagnostic workup, especially when secondary neurologic deterioration begins in the absence of hemorrhagic complications. Regarding therapeutic aspects, once subarachnoid bleeding surrounding the injured vessel is ruled out, more intense medical therapy might be considered, even in large infarctions or in the presence of small aneurysms. Furthermore, TCCD might constitute a useful instrument to assess longitudinally whether the injured vessel maintains its patency in the acute phase.
